# The spectrum of data sharing policies in neuroimaging data repositories

**DOI:** 10.1002/hbm.25803

**Published:** 2022-02-10

**Authors:** Anita S. Jwa, Russell A. Poldrack

**Affiliations:** ^1^ Department of Psychology Stanford University Stanford California USA

**Keywords:** data privacy, data re‐identification, data sharing, data use agreement, neuroethics, neuroimaging

## Abstract

Sharing data is a scientific imperative that accelerates scientific discoveries, reinforces open science inquiry, and allows for efficient use of public investment and research resources. Considering these benefits, data sharing has been widely promoted in diverse fields and neuroscience has been no exception to this movement. For all its promise, however, the sharing of human neuroimaging data raises critical ethical and legal issues, such as data privacy. Recently, the heightened risks to data privacy posed by the rapid advances in artificial intelligence and machine learning techniques have made data sharing more challenging; the regulatory landscape around data sharing has also been evolving rapidly. Here we present an in‐depth ethical and regulatory analysis that examines how neuroimaging data are currently shared against the backdrop of the relevant regulations and policies in the United States and how advanced software tools and algorithms might undermine subjects' privacy in neuroimaging data sharing. The implications of these novel technological threats to privacy in neuroimaging data sharing practices and policies will also be discussed. We then conclude with a proposal for a legal prohibition against malicious use of neuroscience data as a regulatory mechanism to address privacy risks associated with the data while maximizing the benefits of data sharing and open science practice in the field of neuroscience.

## Abstract 2

Sharing data is a scientific imperative that accelerates scientific discoveries, reinforces open science inquiry, and allows for efficient use of public investment and research resources. But for all its promise, the sharing of human neuroimaging data raises critical ethical and legal issues, such as data privacy. Here we present an in‐depth ethical and regulatory analysis that will examine how neuroimaging data are currently shared against the backdrop of the relevant regulations and policies in the United States and how recent advances in software tools and algorithms might undermine subjects' privacy in neuroimaging data sharing.

## 1 INTRODUCTION

Sharing data is an essential aspect of the scientific method (Alter & Gonzalez, 2018; Ross, Iguchi, & Panicker, 2018). It accelerates scientific advancement by creating large data sets through combining multiple data sources and by enabling the investigation of novel hypotheses not conceivable at the time of the initial data collection. Data sharing also makes possible the verification and replication of scientific findings, which reinforces open scientific inquiry and increases public trust in scientific research. It further allows more efficient use of public investment and research resources by preventing redundant data collection. Moreover, data sharing is not just a scientific imperative but also has been suggested to be an ethical duty for researchers to honor the efforts of human research subjects and to maximize the potential benefits of these efforts (Brakewood & Poldrack, 2013).

Considering these benefits, data sharing has been widely endorsed in diverse fields, and neuroscience has been no exception to this movement (Breeze, Poline, & Kennedy, 2012; Choudhury, Fishman, McGowan, & Juengst, 2014; Mennes, Biswal, Castellanos, & Milham, 2013; Poldrack & Gorgolewski, 2014; Poline et al., 2012). The amount of shared neuroimaging data has greatly increased during the last few decades and a number of neuroimaging data sharing initiatives and platforms have been established to promote research on shared data. However, at the same time, data sharing practice in neuroimaging has encountered several challenges, and significant efforts have been made to overcome these challenges.

Initially, technical and infrastructural difficulties presented significant challenges. Given the size of neuroimaging data, building an infrastructure to store and share a massive amount of data poses a substantial barrier in data sharing. It is also crucial to build a field‐wide consensus on how to organize and share neuroimaging data. With the aid of recent technological advancements in data storage, such as commercial cloud computing platforms, along with the exponential increase in computing power, it has become technically straightforward to share large neuroimaging data sets. Efforts to develop a set of community standards on format and structure of neuroimaging datasets (e.g., Brain Imaging Data Structure; Gorgolewski et al., 2016) have also substantially facilitated neuroimaging data sharing. Systems to assign unique identifiers to individual subjects or datasets have also made shared data more easily findable.

Another major challenge lies in motivating researchers to share their data. At the beginning, some researchers expressed concerns that if data were shared, other researchers might analyze the data and publish the results before them or might reveal errors in their initial data analysis (Nichols et al., 2017). Along with the extra costs to prepare and manage shared data, uncertainty around how to credit data sharing in terms of citations and academic promotions has also been another impediment to researchers sharing their data. The pioneering efforts of the fMRI Data Center (fMRIDC), an ambitious neuroimaging data sharing project that began in 1999, involved a burdensome data curation process to those who shared their data and was also criticized by some who objected to data sharing as a prerequisite to publishing in the sponsoring journal (Van Horn & Gazzaniga, 2013). Since then, to address these concerns, data repositories have imposed a grace period before they make data publicly available, which gives data contributors time to publish their research and protects them from being “scooped” by others. In addition, funding agencies have begun to provide financial support for management and sharing of data as part of a research grant. Mechanisms to acknowledge efforts to collect and share data, such as data papers and data citation, have also been introduced in order to encourage the practice of sharing data.

Sharing neuroimaging data also raises important ethical concerns (Brakewood & Poldrack, 2013). If derived from human subjects, the data should be shared in accordance with the ethical principle of protecting human subjects. Yet obtaining fully Informed consent for secondary data analyses is challenging because these analyses might not yet have been developed or even conceived of at the time of initial data collection. Rigorous privacy and security measures should also be in place when sharing data to minimize risks to subjects' privacy and confidentiality of data. Neuroimaging researchers have applied various de‐identification methods, such as removal of direct identifiers (i.e., name and address) from metadata or defacing structural MR images, to protect subjects' privacy (see, e.g., Bischoff‐Grethe et al., 2007).

Unlike the infrastructural and motivational challenges, however, these ethical challenges around data sharing have become increasingly more difficult and complex to navigate, as advances in artificial intelligence and machine learning techniques pose heightened risks to data privacy (Peloquin, DiMaio, Bierer, & Barnes, 2020; Federal Policy for the Protection of Human Subjects, 2018; White, Blok, & Calhoun, 2020). In the United States, federal regulations have provided some guidance on secondary data analysis, but the current regulatory regime mainly relies on researchers' discretion in determining how to, in practice, respect subjects' wishes regarding the future use of their data and protect privacy when data are de‐identified. The emergence of novel software tools and algorithms, such as facial reconstruction techniques (Abramian & Eklund, 2019; Schwarz et al., 2021), is particularly concerning due to their potential ability to invalidate conventional de‐identification methods and to re‐identify subjects in neuroimaging datasets.

As funding agencies have begun to implement their own policies that require investigators to share data collected as part of funded research (National Institutes of Health (NIH), 2020a; National Institute of Mental Health (NIMH), 2019; The Brain Research Through Advancing Innovative Neurotechnologies (BRAIN) Initiative, 2019), sharing data should become increasingly common in the field of neuroimaging, and the body of shared neuroimaging data, including data derived from human subjects, will likely continue to grow substantially. Thus, there is a pressing need to unravel the ethical concerns to promote more responsible sharing of neuroimaging data. Here we present an in‐depth ethical and regulatory analysis that will examine how neuroimaging data are currently shared against the backdrop of the relevant regulations and policies and how advanced software tools and algorithms might undermine subjects' privacy in neuroimaging data sharing. This analysis will first review ethical principles for the protection of human subjects in the context of data sharing and provide an overview of the regulatory landscape regarding data sharing. The regulatory regime on data sharing varies across countries but reviewing all these different regimes is beyond the scope of this article. To provide a more in‐depth analysis, this review will focus on laws and regulations in the United States. Then it will survey the current practice of data sharing in existing neuroimaging data repositories, focusing on restrictions on access to and subsequent use of data in these repositories. Next, novel technological threats of re‐identifying neuroimaging data and the implications these threats could have on data sharing practices and policies will be examined Finally, this article will conclude with proposing a legal prohibition of malicious use of neuroscience data as a regulatory option to address privacy risks associated with the data without unduly limiting the benefits of data sharing and open science practice.

## 2 ETHICAL ISSUES IN NEUROIMAGING DATA SHARING

Sharing human subject data for secondary analysis has been one of the primary topics that arises in discussions concerning the protection of human research subjects. The Ethical Principles and Guidelines for the Protection of Human Subjects of Research (also known as the Belmont Report) laid out three core ethical principles, namely, respect for persons, beneficence, and justice (National Commission for the Protection of Human Subjects of Biomedical and Biobehavioral Research, 1978).

The principle of respect for persons requires human subjects to be treated as autonomous agents and that human subjects be provided special protection should they possess diminished cognitive abilities. Abiding to this principle demands researchers to assure that prospective subjects are capable of making a decision as to whether to participate in a study and that subjects are given sufficient information about the study, such as purpose and procedure of the study, to make informed decisions. Informed consent should include whether data collected from the subjects will be shared for secondary analysis. However, movement to open access and open data sharing calls for a reassessment of the common interpretation of this principle (Ross et al., 2018). In open data sharing, it is difficult to predict and control how neuroimaging data will be analyzed, by whom, and for what purposes once the data are shared through data repositories, and therefore it may not be possible to obtain informed consent, as in a traditional sense, for a specific secondary analysis on the subjects' data. Rather, data sharing requires broad consent that allows a range of possible analyses that are not pre‐specified (45 CFR 46.116d; Bannier et al., 2021).

The principle of beneficence imposes an obligation for researchers to protect subjects by minimizing risks of harm and maximizing potential benefits to the subjects. It requires potential benefits to outweigh any risks of harm in the conducting of a research study. In weighing the risks against the benefits, researchers should consider both the probability and magnitude of harm (Alter & Gonzalez, 2018), and this principle can also be extended to consider the benefits beyond an individual subject participating in a study (Ross et al., 2018). Under this principle, data sharing can be considered an ethical duty for researchers to promote societal good from existing data, such as benefits to public health and welfare, given that subjects are protected from the risks of harm. In the context of neuroimaging data sharing, the primary risks to subjects include the violation of privacy and breach of confidentiality. Privacy and confidentiality differ in a sense that the former applies to persons, whereas the latter applies to personal information and data. Privacy refers to human subjects' interests in controlling “when, how, and to what extent information about them is communicated to others” (Westin, 1967). An extension of privacy, confidentiality pertains to information about subjects disclosed to researchers with the expectation that the information would not be revealed to others (Cooper & McNair, 2015). The duty to maintain confidentiality arises out of researchers' agreement with subjects about how the subjects' information will be handled, managed, and disseminated to prevent unauthorized disclosure of the information. When sharing human subject data, the principle of beneficence obliges researchers to de‐identify data to minimize the risk to privacy and employ rigorous data security measures to prevent breach of confidentiality.

The principle of justice requires fair distribution of the benefits and burdens of human subject research. It primarily relates to equitable selection of subjects for a research study to ensure that subjects do not bear risks to benefit other people. Sharing data could actually promote justice by pooling data from a diverse population, and it facilitates the distribution of the benefits of research findings by increasing the generalizability of these findings.

## 3 OVERVIEW OF REGULATORY LANDSCAPE IN THE UNITED STATES

As a means of implementing the ethical principles of human subject protection in the Belmont Report, a multitude of federal regulations and policies have been stipulated that serve to protect human subjects participating in research studies. This section will review these regulations and policies. It will specifically focus on their application to the sharing of and the secondary analysis of neuroimaging data within the research context in an effort to provide a general overview of the regulatory framework in the United States.

### 3.1 Common Rule

The Department of Health and Human Services (HHS) codified the basic regulations to protect human subjects in research. These regulations, better known as “Common Rule,” have been adopted by many other federal departments and operate as the standard for ethical conduct of government‐funded research (Federal Policy for the Protection of Human Subjects, 2018). The Common Rule was recently revised in 2018 to provide additional regulatory options for conducting research involving human subjects (45 CFR 46.102(e)(1)). For human subject research, the Common Rule requires researchers to obtain informed consent from subjects and the Internal Review Board (IRB) to review and approve research activity.

Analysis of shared data is considered *secondary research*, which refers to research use of information or biospecimens originally collected for research studies other than the proposed one or collected for nonresearch purposes. Under the Common Rule, secondary research that obtains, uses, studies, or analyzes identifiable private information falls under the category of human subject research and is thus subjected to the Common Rule requirements. Identifiable private information is that for which the identity of the subject is, or may readily be, ascertained by the investigator or is associated with the information (45 CFR 46.102(e)(5)). Thus, if shared neuroimaging data contain identifiable information, then investigators conducting research on these data must obtain informed consent from individuals to whom the information pertains and undergo IRB review for the research activity.

Under certain circumstances, secondary research with identifiable private information can be exempted from the Common Rule requirements. This includes when the information is publicly available or when the information is recorded by the investigator in such a manner that the identity of the human subjects cannot be readily ascertained (45 CFR 46.104(d)(4)). The 2018 revision of the rule introduced broad consent as a new option for investigators to conduct secondary research. Broad consent is intended to allow subjects to exercise their autonomy in controlling the use of information pertaining to them while facilitating future research use of the information when the details of such future research have not yet been specified (45 CFR 46.116(d)). If broad consent is obtained at the time of initial collection of identifiable information, storage, management, and use of this information for secondary research are exempted under the Common Rule, and only limited IRB review is required (45 CFR 46.104(d)(7), (8); 45 CFR 46.111(a)(8)). In cases in which these exemptions are not applicable, investigators can still seek waiver of informed consent to conduct the research (45 CFR 46.116(f)(3)).

Unlike secondary use of identifiable private information, research on nonidentifiable private information does not constitute human subject research under the Common Rule. According to a guidance from the HHS Office of Human Research Protections (OHRP) (2008), private information is not individually identifiable when it cannot be linked to specific individuals by an investigator either directly or indirectly through coding systems. For example, coded private information is no longer identifiable, or is anonymized, when a key to decipher the code and enable linkage of the identifying information (e.g., name or social security number) to the private information is destroyed. The guidance further provides that even when the key to the code still exists, secondary research with only coded private information is not considered involving human subjects, if the investigators and the holder of the key to the coded information (e.g., a researcher who collected the information from the individuals) enter into an agreement prohibiting the release of the key to the investigators; if there are IRB‐approved written policies and operating procedures for a repository or data management center that prohibit the release of the key to the investigators; or if there are other legal requirements prohibiting the release of the key to the investigators (Office of Human Research Protections (OHRP), 2008). In most neuroimaging data shared through existing repositories, identifiable information is deleted or replaced with a code (e.g., a number, letter, or symbol) and shared under a data use agreement, which prohibits reestablishing the identity of the subjects. Thus, secondary research on these nonidentifiable neuroimaging data would not be considered research involving human subjects under the OHRP guidance. Yet it is important to note that some IRBs might impose more rigorous standards (e.g., an investigator conducting a study on coded private information may be considered able to ascertain the identity of the subjects if the holder of a key to the code is also listed in the study's IRB protocol), thus researchers should consult with their IRBs when sharing coded private information.

The Common Rule has its own enforcement mechanism for researchers and institutions that failed to meet the requirement for human subject protections, including suspension of research or termination of federal funding (45 CFR §46.123). However, the rule does not provide private cause of action for violation of the regulation, and currently, subjects who experience harm as a result of their participation in a research study are left without any legal remedies (Koch, 2015).^1^

### 3.2 Health Insurance Portability and Accountability Act

The Health Insurance Portability and Accountability Act (HIPAA) (1996) was enacted to improve the efficiency and effectiveness of the healthcare system and includes provisions—the Privacy Rule and Security Rule—to establish national standards to protect medical records and other personal health information. The HIPAA Privacy Rule defines and limits the circumstances in which an individual's protected health information may be used or disclosed by certain covered entities, such as health plans, health care providers, or healthcare clearinghouses (U.S. Department of Health and Human Services (HHS), 2003a). Research institutions, such as a college or university, could also qualify as covered entities, if they have components that perform covered functions (e.g., providing health care at their medical facilities) (U.S. Department of Health and Human Services (HHS), 2008).^2^ As discussed above, most federally funded research involving human subjects is regulated under the Common Rule, which provides protections for the privacy of subjects and for the confidentiality of information collected for a research. The Privacy Rule builds upon these protections, and if a researcher conducting a neuroimaging study is a part of a covered entity, she would also be subjected to the Privacy Rule.

Under the HIPAA, protected health information (PHI) is defined as individually identifiable health information that a covered entity holds or transmits (45 CFR §164.103). Here, individually identifiable health information refers to information related to the individual's past, present, or future physical or mental health/conditions *and* identifies the individual or for which there is a reasonable basis to believe that it could be used to identify the individual (45 CFR §164.103). Individually identifiable health information includes many common identifiers (e.g., name, address, birth date, Social Security number). A covered entity may not use or disclose PHI except in instances in which the Privacy Rule requires or permits it to do so. For example, use and disclosure of PHI for research purposes is permitted with documentation of an alteration (or a waiver) of individual's authorization approved by IRB (45 CFR §164.512(i)). The Privacy Rule also permits the research use of PHI, if certain, specified unique identifiers of individuals have been removed (“limited data set”) and the recipient enters into a data use agreement that promises to safeguard the PHI within the data set (45 CFR §164.514(e)(2), (4)).

However, the Privacy Rule also allows a covered entity to use PHI to create information that is not individually identifiable (45 CFR §164.502(d)), and the use or disclosure of this de‐identified information is no longer regulated under the HIPAA (45 CFR §164.514(a)). Unlike the Common Rule that provides only a general definition of nonidentifiable private information, HIPAA sets out two specific standards to determine de‐identification of PHI. One is a formal determination by a qualified statistician that the risk is extremely low that the information could be used—alone or in combination with other reasonably available information—to identify an individual who is a subject of the information (45 CFR §164.514(b)(1)). The other, known as the safe harbor method, entails the removal of 18 unique identifiers from PHI provided that the covered entity has no actual knowledge that the remaining information could be used to identify the individual (45 CFR §164.514(b)(2)). This method differs from anonymization of data, which requires a code or other link to trace the information back to specific subjects to be destroyed. Under the HIPAA regulation, de‐identified neuroimaging data that do not contain these identifiers can be shared and analyzed without restrictions. The safe harbor method has been widely accepted and used by researchers, even if they are not subjected to HIPAA, as a conventional way to de‐identify data.

The HIPAA Security Rule has stipulated security standards for the protection of PHI that is held or transferred in electronic form (e‐PHI) by covered entities. These standards are intended to operationalize the protections provided in the Privacy Rule (U.S. Department of Health and Human Services (HHS), 2003b). The Security Rule requires covered entities to implement reasonable and appropriate administrative, technical, and physical safeguards to ensure the confidentiality, integrity, and availability of e‐PHI (45 CFR §164.304, 306). For example, covered entities must perform a risk analysis to evaluate potential security risks to e‐PHI and to put appropriate security measures in place to prevent unauthorized disclosure of e‐PHI (45 CFR § 164.308(a)(1)(ii)(A)). Again, the Security Rule does not apply when covered entities create, receive, maintain, or transmit de‐identified PHI.

HIPAA imposes substantial financial penalties to covered entities that failed to comply with its regulations, but similar to the Common Rule, HIPAA does not provide a private cause of action that allows for an individual to sue a covered entity for violation of the HIPAA (U.S. Department of Health and Human Services (HHS), 2013).^3^ Nevertheless, an individual can file a health information privacy complaint to the Office of Civil Rights (OCR) (45 CFR §160.306.). The OCR will conduct an investigation upon receiving the complaint and issue a letter regarding the resolution of the complaint. If a covered entity was found to have violated HIPAA, it must voluntarily comply with the HIPAA rules, take corrective action, or agree to a settlement (U.S. Department of Health and Human Services (HHS), 2017a). A covered entity that fails to take satisfactory action to resolve the matter may be subject to civil monetary penalties or criminal prosecution (U.S. Department of Health and Human Services (HHS), 2017b).

### 3.3 The NIH data sharing policy

The National Institutes of Health (NIH), one of the major federal funding agencies for biomedical research in the United States, has published its own policy to promote the sharing of data, including neuroimaging data, generated from research funded or conducted by the NIH. This policy, which will be effective from January 2023, requires researchers applying for NIH funding to submit a Data Management and Sharing Plan (DMS plan) for any NIH‐funded or conducted research that will generate scientific data (National Institutes of Health (NIH), 2020a). While acknowledging the importance and benefits of data sharing, the NIH has emphasized the protection of rights and privacy of human subjects who have participated in NIH‐funded research.

The policy does not introduce new additional requirements regarding for the protection of human subject data beyond existing laws and regulations (e.g., the Common Rule or Certificates of Confidentiality^4^), but researchers must outline in their DMS plan how privacy, rights, and confidentiality of human subjects will be protected. The policy provides three concepts to consider in sharing human subject data: (1) how to address data management and sharing in the informed consent process, (2) whether there are any limitations on subsequent use of data should be communicated to those individuals or entities preserving and sharing the data, and (3) even when data have been de‐identified and lack explicit limitations on subsequent use, whether access to these data should still be controlled (National Institutes of Health (NIH), 2020a). Yet, it is important to note that this policy does not preclude the open sharing of data from human subjects in ways that are consistent with consent practices, established norms, and applicable laws.

The policy also provides a guidance for researchers on how to choose a proper data repository by delineating the desirable characteristics of a repository, including that having security measures in place to prevent unauthorized access to, modification of, or release of sensitive data and having the capabilities to ensure confidentiality of data (National Institutes of Health (NIH), 2020b). In specific regard to repositories storing human subject data, the policy lists additional considerations, for example, whether a repository restricts access to and use of data consistent with subjects' consent; communicates and enforces data use restrictions, such as preventing re‐identification or redistribution to unauthorized users; and implements appropriate approaches (e.g., tiered access, credentialing of data users, security safeguards against potential breaches) to protect human subject data from inappropriate access (National Institutes of Health (NIH), 2020b).

### 3.4 The NIMH data sharing policy

In 2019, the National Institute of Mental Health (NIMH), an NIH institute for research on mental disorders, published a Notice of Data Sharing Policy (National Institute of Mental Health (NIMH), 2019). It requires researchers who are funded by NIMH to deposit their data, including neuroimaging data, to the NIMH Data Archive (NDA). All applications involving human subjects that are submitted to or referred to NIMH after January 1, 2020 are expected to include a Resource Sharing Plan as part of the application, except applications for Fellowship (F), Research Career Development (K), Training (T), Small Business (SBIR/STTR), Small Grants (R03), Education (R25) and Awards related to AIDS applications A single NDA collection containing the data associated with each grant award will be established, and data from the award are expected to be submitted every 6 months. Submitted data will generally be held in a private enclave until papers using the data have been accepted for publication or until the end of the award period (including the first no‐cost extension), whichever occurs first. In addition to submitting the data associated with a grant award, researchers are expected to separately submit to the NDA the specific data that were used for each resulting publication by creating an NDA Study to promote rigor and reproducibility of research. The NIMH also strongly encourages researchers to deposit their data from the NIMH‐funded research to other appropriate repositories, such as the Brain Research Through Advancing Innovative Neurotechnologies (BRAIN) Initiative data archives.

### 3.5 The BRAIN Initiative data sharing policy

Particularly relevant to our analysis, the BRAIN Initiative, a large‐scale trans‐agency effort to support research on human brain and function, also released a notice of its data sharing policy in 2019 (The Brain Research Through Advancing Innovative Neurotechnologies (BRAIN) Initiative, 2019). For all BRAIN Initiative applications submitted after March 1, 2020, applicants are required to include a Resource Sharing Plan as part of the application and to submit their data to one of the designated archives established to hold data collected as part of BRAIN Initiative‐funded research. These archives include The Neuroscience Multiomic Data Archive (nemoarchive.org) to hold data from ‐omics experiments; The Brain Image Library (brainimagelibrary.org) to hold microscopy data; Data Archive for the BRAIN Initiative (dabi.loni.usc.edu) to hold data related to human electrophysiology experiments; OpenNeuro (openneuro.org) to hold neuroimaging data that adhere to the Brain Imaging Data Structure (BIDS) standard; Block and Object Storage Service (bossdb.org) to hold electron microscopy data. Similar to the NIMH data sharing policy, applicants are expected to submit data to the archives every 6 months, which will be held in a private enclave until papers using the data have been published or until the end of the award period. For research that involves human subjects, the Resource Sharing Plan should have a description of whether and how the consent obtained from the subjects to collect data will limit future secondary research that can be done with the data. The notice further encourages applicants to use consent that allows for broad data sharing whenever possible.

The review of regulatory protections for human subjects indicates that research on shared neuroimaging data that contain identifiable private information be subject to the Common Rule requirements. If a researcher or a research unit is part of a covered entity under the HIPAA, sharing neuroimaging data with PHI should also comply with the HIPAA Privacy and Security Rule. However, neuroimaging data that have had identifiable information removed or coded in a way that makes the data nonidentifiable under the Common Rule or that are de‐identified under the HIPAA can be shared without restrictions under these regulations. Funding agencies' data sharing policy also requires researchers to consider how to protect human subjects' privacy and the confidentiality of data derived from human subjects and to ensure that researchers conducting secondary analyses should abide by any additional limitations on subsequent use of data.

## 4 DATA SHARING PRACTICE IN EXISTING NEUROIMAGING DATA REPOSITORIES

Laws and regulations have stipulated general compliance standards for adequate data protection but examining how neuroimaging data are currently shared in data repositories can help us to understand the actual practice of data sharing in the field of neuroimaging. This section will survey data sharing practices in existing neuroimaging data repositories, focusing on restrictions on access to and subsequent use of data shared through these repositories.

### 4.1 What types of neuroimaging data are shared?

Existing neuroimaging data sharing initiatives or platforms offer different types of neuroimaging data, which involve varying levels of privacy risks. Some repositories only share peak coordinates of brain regions that showed statistically significant effects in published neuroimaging studies (e.g., The BrainMap project (Vanasse et al., 2018) and the Neurosynth project (https://neurosynth.org)). These coordinate‐based data can be used to generate maps that aim to reconstruct the original activation map, though the sparse nature of the coordinate‐based data leads to substantial loss of information compared to the original (Poldrack & Gorgolewski, 2014). The next level is sharing unthresholded maps or network maps (e.g., Neurovault; Gorgolewski et al., 2016), which allows researchers to both examine results of the original study and conduct meta‐analysis on subthreshold effects in brain regions beyond the peak coordinates reported in publications. The Brain Analysis Library of Spatial maps and Atlases (balsa.wustl.edu) is another repository that shares extensively analyzed spatial maps associated with specific published studies. Research on coordinate‐based data or statistical/spatial maps does not pose significant privacy risks because these maps only provide group‐level information. However, sharing unprocessed or minimally preprocessed individual‐level data, such as NIfTI or DICOM files, could provide greater potential by enabling researchers not only to reanalyze the initial results but also to investigate new hypotheses beyond the ones tested in the original study. OpenNeuro (openneuro.org), International Neuroimaging Data‐Sharing Initiative (INDI) (fcon_1000.projects.nitrc.org), Human Connectome Project (HCP) (Van Essen et al., 2013), and Alzheimer's Disease Neuroimaging Initiative (ADNI) (Jack Jr. et al., 2008) are some of the repositories that store and share raw (or preprocessed) individual structural, resting‐state, or functional MRI data.

### 4.2 How are neuroimaging data shared?

The survey of existing neuroimaging data repositories shows a wide spectrum of data sharing practices. Some repositories offer fully open sharing without any limitations attached, whereas others impose certain restrictions on access to and subsequent use of data under their data sharing policy or data use agreement.

#### 4.2.1 Repositories offer fully open sharing

Most repositories that support fully open sharing host neuroimaging data under certain Creative Commons (CC) licenses. For example, OpenNeuro (openneuro.org) requires researchers to agree that raw individual‐level data will be shared under Creative Commons CC0 license by waiving copyright and all other related rights to the data with a grace period of 36 months from the upload of the data. The CC0 license essentially puts the data in public domain (creativecommons.org/publicdomain/zero/1.0/), and thus, there is no restriction on the access and further use of data shared through OpenNeuro after the grace period. To protect subjects' privacy, researchers contributing data to OpenNeuro are also required to de‐identify the data by deleting the unique identifiers as defined under the HIPAA, including facial features on structural images, and have ethical permission from their own institution to publicly share data. Other open sharing initiatives or platforms, such as 1,000 Functional Connectomes Project (FCP) (Biswal et al., 2010) and International Neuroimaging Data‐Sharing Initiative (INDI) (fcon_1000.projects.nitrc.org), both prospectively and retrospectively, share data under Creative Commons, Attribution‐NonCommercial‐ShareAlike License (creativecommons.org/licenses/by-nc-sa/4.0/). It is free to copy, redistribute, and adapt data, but unlike the CC0 license, data can be shared only for noncommercial research purposes, and users must give appropriate credit for the shared data.^5^ The INDI also has detailed guidelines for researchers to follow when preparing their data for sharing (International Neuroimaging Data‐Sharing Initiative, n.d.). Specifically, it requires that datasets do not contain any identifiable information in the images and image‐headers to ensure HIPAA‐compliant de‐identification. Facial features should also be removed from any high‐resolution images and each subject's ID must be changed to an anonymized numeric value provided by the INDI.

#### 4.2.2 Repositories have a data sharing policy or data use agreement

Some neuroimaging data repositories require researchers who want to share their data or use shared data for secondary analysis to explicitly agree with their data sharing policy or data use agreements, which include various restrictions on data access and future use of data. This section will review these data sharing policies and data use agreements in select neuroimaging repositories.

##### The Open Access Series of Imaging Studies

The Open Access Series of Imaging Studies (OASIS) project (oasis-brains.org) archives neuroimaging datasets on cognitively normal and demented subjects (with mild to moderate Alzheimer's disease) that are freely available to the scientific community with relatively minimal requirements under its data use agreement. The data shared through the OASIS are de‐identified, but some data sets do contain nondefaced structural images (Marcus, Fotenos, Csernansky, Morris, & Buckner, 2010). The data use agreement (DUA) of the OASIS project sets out terms including that users should not attempt to establish the identity of human subjects, and should acknowledge the use of OASIS data when publicly presenting any results or algorithms that benefited from the data (The Open Access Series of Imaging Studies, n.d.). Users could also be requested to provide updates on research use of data. Failure to abide by these terms will result in termination of the right to access and use OASIS data.

##### The Human Connectome Project

The NIH Human Connectome Project (HCP) is an effort to acquire and share data on the structural and functional connectivity of the brain to map the neural pathways that underlie human brain function. It established two research consortia to achieve this goal, the Washington University in St. Louis, University of Minnesota, and Oxford University project (Van Essen et al., 2013; humanconnectome.org) and the Harvard/Massachusetts General Hospital – University of Southern California project (humanconnectomeproject.org). These two consortia have different DUA for the use and sharing of data collected.

The WU/Minn HCP consortium developed an initial study, now known as the Young Adult HCP project, which shared data from about 1,200 individuals. Subsequent “HCP‐style” projects include the Lifespan HCP project in Development and in Aging and a number of disease‐specific Connectome projects.^6^ The Young Adult HCP data are released through the ConnectomeDB (db.humanconnectome.org). Because this subproject includes subjects who are twins and siblings from extended families located in a limited geographical area, the potential for re‐identification is increased. For this reason, the project implemented a two‐tiered approach that distinguishes data that can be openly shared and those for which access should be restricted. Open access data essentially include all image data and most of the behavioral data stored in the database, and restricted data include information such as family structure (e.g., twin status) and age that could be used to re‐identify subjects if combined with open access data. Open access data are not considered fully de‐identified, as certain combinations of HCP restricted data might allow identification of individuals (WU‐Minn HCP Consortium, n.d.‐a), although all open access HCP structural scans are defaced (WU‐Minn HCP Consortium, n.d.‐b). Along with the requirement of preventing users from re‐identifying and contacting human subjects, the Data Use Terms for open access data emphasizes that the code linking data to PHI or any additional information about individual subjects will not be provided under any circumstances (WU‐Minn HCP Consortium, 2013). It further specifies that users should comply with all relevant rules and regulations regarding the protection of human subjects (e.g., proposed research should be approved or exempted by IRB or Ethics Committee at the investigator's institution). Open access data, and any derived data, may be redistributed under the same terms, and users should acknowledge the WU/Minn HCP when publicly presenting results gained from the data. Again, failure to comply with the terms will cause termination of access to data. In order to obtain access to restricted data, users are required to abide by additional terms to protect subjects' privacy, such as prohibition of further distribution or sharing of data, secured access to data, and restrictions on the publication of data (WU‐Minn HCP Consortium, 2016).

The Harvard/MGH‐USC HCP consortium also asks users to abide by its DUA in order to obtain access to shared data (Harvard/MGH‐USC HCP consortium, n.d.). Its DUA prohibits users from re‐identifying subjects and disclosing data beyond the uses outlined in the DUA (e.g., purposes of scientific investigation, teaching, or the planning of clinical research studies) or delineated in the application by the users. It also requires users to employ appropriate administrative, physical, and technical security measures to prevent use or disclosure of the data other than as provided in the DUA and the application. Users should report any event of unauthorized use or disclosure, and they are expected to acknowledge and cite the HCP as the source of data in the publications.

##### The National Institute of Mental Health Data Archive

The National Institute of Mental Health (NIMH) Data Archive (NDA) (nda.nih.gov) was established to store and share data related to autism research but has evolved into an informatics platform that houses data collected as part of NIMH funded research. The archive also stores data from mental health research initiatives, such as the Adolescent Brain Cognitive Development (ABCD) Study (abcdstudy.org) and the Connectome Coordination Facility (CCF) (humanconnectome.org/lifespan-studies), and operates as an overarching database to provide a single process for gaining access to the data in these repositories. The NDA Data Sharing Terms and Conditions sets forth the expectations and procedures that investigators who want to submit their data to the NDA, which includes that all data submitted to the NDA should be de‐identified, although it does not explicitly require defacing of structural scans, and should be collected from subjects who consented to broadly share their data for research use (National Institute of Mental Health Data Archive (NDA), 2020). Users requesting access to NDA data are required to agree with terms and conditions laid out in its Data Use Certificate (DUC). (National Institute of Mental Health Data Archive (NDA), 2021). The DUC allows access to data only for research purposes and prohibits re‐identification of subjects or any further re‐distribution of data. Users should also comply with regulatory and institutional requirements for protection of human subjects and safeguard data with security best practices to prevent unauthorized access to data. Moreover, they are required to provide an annual summary of research accomplishments achieved by using the NDA data and to report publications by creating an NDA Study that associates the publications and results with the underlying data in the NDA.

##### Alzheimer's Disease Neuroimaging Initiative

Alzheimer's Disease Neuroimaging Initiative (ADNI) is a longitudinal multicenter project that aims to develop clinical, imaging, genetic, and biochemical biomarkers for the early detection and tracking of Alzheimer's disease (Jack Jr. et al., 2008). For future sharing and secondary use of data, its data sharing policy requires an informed consent form used in each of the ADNI sites to include a statement that data collected from subjects will be de‐identified and shared with ADNI members and the scientific community for research purposes (Alzheimer's Disease Neuroimaging Initiative (ADNI), 2016a). Investigators who want to gain access to data should first submit an application to the ADNI Data and Publication Committee (DPC). If applicants are qualified as members of the scientific community described in the consent form, the ADNI provides full, open access to all ADNI imaging and clinical data, which are limited data sets as defined under HIPAA, to applicants who agree to the conditions in the ADNI Data Use Agreement (Alzheimer's Disease Neuroimaging Initiative (ADNI), 2016b). After access to data is granted, applicants will receive annual requests to update the application information in addition to queries about manuscripts that resulted from research on shared data (e.g., title of manuscripts, information on authors of manuscripts, and status of each manuscript in development). Similar to the DUAs in other repositories, the ADNI DUA prohibits users from establishing the identity of subjects or contacting the subjects. No future disclosure of data beyond the uses outlined in the DUA and the application are allowed. Users should ensure to employ appropriate administrative, physical, and technical safeguards to prevent use or disclosure of the data other than as provided in the DUA. The DUA also requires acknowledging the ADNI as the data and funding source, but it further asks users to submit all manuscripts prior to submitting to a journal. The ADNI DPC will conduct an administrative review to confirm that the manuscript acknowledged the ADNI as outlined in the DUA. Finally, users should report any use or disclosure not provided for by the DUA, and noncompliance with the required updates will jeopardize future access to ADNI data.

#### 4.2.3 Summary

Table 1 summarizes the actual practice of data sharing in existing neuroimaging data repositories surveyed above. Each row of the table represents a restriction or requirement implemented by data repositories (mostly corresponding to one provision in a data sharing policy or DUA). In general, the level of restrictions in these repositories varies depending on the sensitivity of data and other relevant factors, such as whether original contributors still retain some control over shared data (e.g., by employing strict acknowledgment requirement) or whether a federal agency (e.g., NIH or FDA) is involved as a governing body of a repository.

**TABLE 1:** Current practice of data sharing in existing neuroimaging data repositories

	Fully open sharing		Sharing with data sharing policy or data use agreement					
	OpenNeuro	INDI/FCP	OASIS project	HCP			NDA	ADNI
				WU/Minn HCP		Harvard/MGH‐USC HCP		
				Open access data	Restricted data			
De‐identification of data for sharing	✔	✔	✔	✔	✔	✔	✔	✔
Prohibition on re‐identifying subjects			✔	✔	✔	✔	✔	✔
Limitations on further disclosure or use of data					✔	✔	✔	✔
Security measures in place					✔	✔	✔	✔
Acknowledgement of data repository as data source		✔	✔	✔	✔	✔	✔	✔^a^
Report research use of data upon request			✔			✔	✔	✔
Report of violation						✔	✔	✔

*Note*: This table is based on requirements or restrictions *explicitly* stated in the data sharing policy or data use agreement.

^a^ Additional requirement of review of manuscripts by the ADNI Data and Publication Committee prior to journal submission.

Regardless of whether repositories provide fully open sharing or sharing with data sharing policies or DUA, all of them require shared data to be de‐identified. Every repository that shares neuroimaging data under data use agreement has a provision that prohibit re‐identifying or contacting data subjects, which makes shared data nonidentifiable under the Common Rule and thus, secondary analysis on the data is considered research not involving human subjects. Except data repositories that provide fully open sharing, only the OASIS Project and the WU/Minn HCP's DUA for Open Access Data do not explicitly impose limitations on further disclosure or use of data and do not require security measures. In addition, all the repositories that share data under a DUA, except the WU/Minn HCP (both for Open Access and Restricted Data), request researchers to report research use of data. Reporting violation of terms of a data sharing policy or a DUA is required by the Harvard/MGH‐USC HCP, the NDA, and the ADNI.

#### 4.2.4 Alternative approaches

Some research initiatives and databases have attempted to develop an alternative approach to sharing human subject data to better protect subjects' privacy or to respect subjects' autonomy on how their data should be used. For example, instead of sharing raw individual‐level data, the Enhancing Neuroimaging Genetics through Meta‐analysis (ENIGMA) provides analysis protocols to run at a local site and perform meta‐analysis on results (e.g., summary statistics) returned from the local sites (Thompson et al., 2014; Thompson et al., 2020). The Collaborative Informatics and Neuroimaging Suite Toolkit for Anonymous Computation (COINSTAC) is another large‐scale research consortium that offers tools for decentralized analysis which allows researchers to conduct both meta‐ and mega‐analysis without pooling the data at one central place (Ming et al., 2017; Plis et al., 2016). Groups of users run common analyses on their local sites using their own data and the results of these analyses are then synchronized to the cloud and undergo aggregate analyses processes using all local data. The federated computing of COINSTAC also provides heightened privacy protection using advanced statistical algorithms, such as differential privacy, and this model may open up a way to gain access to data that researchers are unable to share due to local regulatory restrictions (Ming et al., 2017; White et al., 2020). However, it is important to note that federated computing could make data sharing more burdensome because it would require significant synchronized agreement efforts among all the sites to run the analysis locally or to change the protocol.

OpenHumans (openhumans.org) is a database that allows willing individuals or communities to share their own personal data for research purposes. Although most data currently shared in this database are genetic, demographic, or other personal data (e.g., social networking service data or data from digital healthcare apps), it may provide a useful reference for a potential new form of sharing neuroimaging data. In OpenHumans, individual contributors can opt in to certain activities, such as a research study, listed in the database. The recipients of data, investigators of the activities, are required to follow OpenHumans Activity Guidelines (OpenHumans, n.d.). Under the guidelines, the investigators should provide information on how data will be managed, such as whether data is de‐identified or potentially identifiable, sensitivity of data, or other relevant privacy and security issues, to individuals to whom data pertains or to the IRB/ethics board. The investigators should also explain how they will handle data if an individual contributor decides not to participate in the activity any longer (e.g., deleting data). Some individuals make their data publicly available under the CC0 license, and investigators using these public data are subject to the requirements under OpenHumans Public Data Guidelines, which include preventing subject re‐identification, acknowledging the data source, and providing a reference to these guidelines in any case of re‐distributing the data (OpenHumans, n.d.).

## 5 NOVEL TECHNOLOGICAL THREATS TO DATA PRIVACY IN NEUROIMAGING DATA SHARING

The potential risks of data sharing to privacy and confidentiality have long been acknowledged among researchers. Yet it has been widely accepted that data sharing in research contexts is possible without compromising data privacy and confidentiality by adopting relatively simple data redaction methods and security measures. Removing specific information that can identify individual subjects from metadata has also been a common method of ensuring privacy protection. In neuroimaging studies, some researchers have blurred or removed individual subject's facial features from structural MR images (Bischoff‐Grethe et al., 2007; Milchenko & Marcus, 2013). Physically securing devices that store data (i.e., a computer or disks) and controlling access to data with password protection have also been perceived as reasonable practices of data security.

However, the emergence of advanced software tools and algorithms poses novel threats to data privacy because of their ability to re‐identify subjects in neuroimaging datasets that have been thought to be de‐identified. One of the well‐known techniques reported to be able to invalidate conventional de‐identification methods is facial reconstruction. In their 2019 study, Schwarz and colleagues showed that when only metadata are de‐identified while facial features in structural images remain intact, it is possible to reconstruct individual subject's faces and re‐identify them by matching the reconstructed faces with photos from social media or other resources using a facial recognition software (Schwarz et al., 2019). Other studies also have shown that even when neuroimaging data are defaced, it is still possible to reconstruct facial images. For example, Abramian and Eklund reported that generative adversarial networks (GANs), which is a popular tool for realistic image synthesis, can reconstruct facial features from defaced structural MR image slices (Abramian & Eklund, 2019). The GANs‐based algorithm developed in this study was able to convincingly restore face‐blurred image slices, although it showed limited success for image slices in which facial features had been deleted. Re‐identifying subjects with this technique could lead to a disclosure of related data or other information, such as other nonimaging data (e.g., cognitive scores), diagnosis or biomarkers of a disease, or participation in studies or clinical trials; however, the ability to reconstruct individual faces sufficiently well for re‐identification has yet to be demonstrated, and it seems unlikely that the details of facial structure necessary for re‐identification (e.g., eye spacing) could be reconstructed from skull structure after defacing.

Functional MRI fingerprinting, a technique that extracts individually unique patterns of functional images, can also be used to infer the identity of subjects. Studies have reported that identification using these brain signatures or fingerprints generates highly accurate results (Finn et al., 2017; Ravindra, Drineas, & Grama, 2021; Vanderwal et al., 2017). Ravindra and Grama demonstrated that if one publicly available dataset is re‐identified, then the individually unique functional signature can be matched across other datasets, which contain scans of the same subjects, leading to disclosure of the subjects' sensitive information in these datasets (Ravindra & Grama, 2019). Given the recent increase in sharing of multi‐modal datasets, as in HCP or ENIGMA that share genetic data along with neuroimaging data, MRI fingerprinting could potentially heighten the risks of revealing sensitive information about individual subjects. Combining multiple databases and collecting data from a longitudinal study could further exacerbate these risks to privacy. However, the accuracy of such methods decreases substantially when applied to larger datasets (Waller et al., 2017), suggesting that it may not be feasible to accurately re‐identify individuals in real‐world scenarios.

In addition, there have been significant advances in more generic ways to re‐identify subjects based on nonimaging data, using statistical modeling. For example, one study showed that by using a generative graphical model, individuals can be accurately re‐identified with high likelihood even from heavily incomplete de‐identified socio‐demographic, survey, and health datasets (Rocher, Hendrickx, & de Montjoye, 2019).

## 6 IMPLICATIONS OF NOVEL THREATS TO PRIVACY IN NEUROIMAGING DATA SHARING PRACTICES AND POLICIES

Conventional de‐identification methods, such as removal of direct identifiers in metadata or removing facial features in neuroimaging data, have been thought to be sufficient to protect subjects' privacy. In the United States, the regulatory protections for human subjects do not apply to de‐identified neuroimaging data (Rothstein, 2010). Secondary analysis on de‐identified neuroimaging data is not considered human subject research under the Common Rule, and thus, investigators are not required to comply with its requirements, informed consent and IRB review. De‐identified data also do not constitute protected health information under the HIPAA. In other words, once data are de‐identified, the current US regulatory system primarily relies on researchers to make ethical and judicious decisions on how and where to share their data (Ross et al., 2018). The basic assumption here is that researchers follow ethical principles and community norms in selecting data repositories and limiting subsequent use of data.

However, recent studies using novel tools and algorithms, such as facial reconstruction or MRI fingerprinting, have shown that any rigorously de‐identified data sets still contain some risks of re‐identification. To defend against this re‐identification attack, researchers have been developing ways to counteract these tools and algorithms—for example, a new more effective defacing technique for structural images (Schwarz et al., 2021) or removal of signature patterns in functional images. Yet given the unprecedented pace of advancement in artificial intelligence and machine learning algorithms, it would be nearly impossible to ensure complete anonymization of data.

One might argue that there is a need for a more rigorous data protection regime to protect research subjects' privacy against these novel technological threats. Yet blanket regulation on data sharing might impose a greater burden on researchers who want to share low‐risk data yet still provide insufficient protection for extremely sensitive data. Moreover, the fact that it is *possible* to re‐identify subjects does not necessarily mean that it is *likely* to happen, especially given the current premature state of technology. The results from the recent studies might seem alarming, but these software tools and algorithms are still at an exploratory stage and have only been used for demonstration purposes. It should also be noted that re‐identification per se can rarely cause harm to research subjects—that would require the actual misuse of data. Subjects would suffer harms only when sensitive information disclosed from re‐identification is used to jeopardize the subjects' interests, as in the cases wherein a subject's disease state or biomarker derived from neuroimaging data is used to damage the subject's reputation or to put subjects' interests in peril in employment or health insurance contexts.

Unfounded fear or hype around what can be inferred from neuroimaging data could also dissuade not only potential subjects but also researchers from participating in neuroimaging research or sharing neuroimaging data. Choudhury and colleagues have argued that given the popular interest in neuroimaging that is “riddled with metaphors about ‘mind‐reading’ capacities of neuroimaging and essentialistic hype about brain scans and personal identity,” it is important to endorse critical understanding among the general public on the limits to what information about an individual can be inferred from neuroimaging data (Choudhury et al., 2014). The most salient risk to subjects that would occur through re‐identification would be the use of those data to derive health‐related information, such as the risk of developing a brain disorder such as Alzheimer's disease, or through the prediction of future behavior, such as criminal behavior. Thus, a fair assessment of the accuracy of brain imaging for such predictions is essential to realistically understanding the potential risks of re‐identification. At present, there is reason to doubt that current neuroimaging methods will provide highly accurate prediction of future outcomes. First, there is evidence that reported predictive accuracy rates in the literature may be systematically inflated, due to the use of small samples along with analytic flexibility (Poldrack, Huckins, & Varoquaux, 2020; Varoquaux, 2018). In addition, the real‐world accuracy of prediction for rare outcomes (such as specific diseases or behavioral outcomes such as criminality) is heavily overestimated in studies that compare groups of equal sizes (Poldrack et al., 2020); measures that appear to be highly accurate in these settings are almost guaranteed to have very high rates of false positives when applied to the prediction of rare outcomes, or to have very low sensitivity if false positive rates are controlled. Thus, while it is impossible to predict the utility of future measures, the prediction of relatively rare outcomes is a fundamentally difficult problem; there is currently insufficient evidence to suggest that neuroimaging data can provide powerful predictions of future outcomes that could be used to discriminate against individuals.

Regardless of the level of risk, a more cautious approach needs to be developed to promote responsible sharing of neuroimaging data—for example, tiered control of data carefully calibrated to a realistic assessment of privacy risks (Ross et al., 2018). There has been a lack of regulatory oversight of data repositories, but the survey of data sharing practices in neuroimaging repositories showed that the repositories have already developed varying levels of access control and limitations on subsequent use of data, even de‐identified, depending on the nature of data and the risks of harm associated with re‐identification of data, among other relevant factors. Except in cases in which funding agencies require deposition of data in certain designated repositories, it can thus be said that the current ecosystem of neuroimaging data sharing offers a variety of options for researchers to share their data, including the alternative approaches of data sharing (e.g., CONISTAC or OpenHumans).

As noted above, the decision on how to share data is left to the discretion of researchers, but the development of general guidelines, such as the supplementary information to the NIH data sharing policy on desirable characters of data repositories (National Institutes of Health (NIH), 2020b), would prove extremely valuable. Some of the considerations that should be included in these guidelines are the use of a consent mechanism that enables broad open sharing of data while ensuring adequate protection for data subject's privacy (Bannier et al., 2021) and the defacing of structural MR images—even if it is not required by a data repository—unless intact facial features are essential for the purpose of a research project. Understanding different levels of openness across data sharing policies of repositories when sharing sensitive data would also be useful in determining where and how to share data. These guidelines could also in turn inform neuroimaging repositories of the need to update their data sharing policies or data use agreements to better protect human subjects.

## 7 TOWARD THE ULTIMATE PROTECTION AGAINST RE‐IDENTIFICATION RISKS IN NEUROIMAGING DATA SHARING

Employing cutting‐edge de‐identification methods would substantially reduce the risk of re‐identification. Controlling access to and subsequent use of neuroimaging data calibrated with the sensitivity of the data would further minimize the potential harm to subjects' privacy. Nevertheless, even with the best available privacy and security measures, it would be impossible to completely eliminate the risks of re‐identification. If this remaining risk, however unlikely, were to materialize, it could potentially result in significant harm to subjects in some cases. Here we propose that this potential harm of re‐identification would be best addressed by introducing regulatory safeguards to prevent the misuse of information derived from neuroscience data, such as neuroimaging data, against the data subject.

As reviewed earlier, the enforcement mechanisms in the Common Rule and HIPAA do not provide proper legal ramifications for individuals who experience harm resulting from their participation in a research study, such as private cause of action regarding research‐related harms. Given this lack of recourse on the side of research subjects, it is also not clear how to address potential harm to subjects caused by disclosure of research data in the context of data sharing under the current regulatory regime. In fact, the issue of using personal and sensitive information against individuals to whom the information pertains has received close attention from policy makers, and regulatory protections have been enacted to tackle this issue in other contexts. The most relevant example is the Genetic Information Non‐discrimination Act (GINA) (National Human Genome Research Institute, 2008). The GINA prohibits discrimination on the basis of genetic information in the context of health insurance and employment.^7^ Under GINA, it is unlawful for health insurers and employers to request, require, or purchase genetic information with respect to an (potential) insured individual or employee. However, it is still possible that they might inadvertently obtain the genetic information of an insurance applicant or an employee. Given that it is not feasible to completely control the risk of disclosure, the protection under GINA is targeted at forbidding health insurers and employers from using one's genetic information for discriminative purposes rather than restraining them from having knowledge on the genetic information altogether (42 U.S.C. § 2000ff‐l(b)(1)‐(6), (c)).

There have been criticisms of GINA, such as its limited scope that only covers health insurance—leaving out other types of insurance (e.g., life insurance and long‐term care insurance), but it laid the groundwork to promote obtaining genetic testing or participating in genetic research by alleviating the public's fear of genetic discrimination. Adopting a regulatory scheme against malicious use of research data, similar to what GINA outlines, would relieve concerns around remaining risks of re‐identification in the context of data sharing and offer ultimate protection for potential harm associated with these risks. Along with sound privacy and security measures, such a regulatory scheme—“The Neuroscience Information Non‐Discrimination Act”—would enable us to chart a path to protect data privacy without unduly undermining the substantial benefits of data sharing to human health.^8^

## 8 CONCLUSION

Neuroimaging is a scientific field that has greatly benefited from data sharing. Data sharing has been largely endorsed by researchers and neuroimaging studies on shared data have resulted in new scientific discoveries that deepen our understanding of the human brain and its functions. Yet for all its benefits, sharing neuroimaging data entails critical ethical and legal concerns, particularly regarding the privacy of subjects and confidentiality of data. In the United States, a multitude of federal and state regulations and policies provide protections for human subjects' identities and other sensitive information, including de‐identification of data. However, recent advancements in software tools and algorithms to invalidate conventional de‐identification methods pose heightened privacy risks in data sharing. Blanket regulations on data sharing could unduly hamper open science practice, so we should take a more cautious approach based on a realistic risk assessment, such as tiered control over data sharing based on privacy risks. In fact, existing neuroimaging databases have already developed varying levels of access control and limitations on the use of data depending on the sensitivity of the data. But given that it is currently under researchers' discretion to decide where to share data, it would be desirable for researchers to have some guidelines to adopt that promote more responsible sharing of neuroimaging data. Along with these guidelines, the remaining risks of re‐identification could be addressed by introducing a regulatory safeguard against misuse of neuroscience information, similar to the safeguard provided in the Genetic Information Non‐Discrimination Act.

## CONFLICT OF INTEREST

The authors declare no potential conflict of interest.

## Data Availability

No new data were created or analyzed to support this study.

## Endnotes

^1^
The United States did not sign on to the 7th edition of the Declaration of Helsinki that has a new general principle regarding the compensation for research‐related injuries; “[a]ppropriate compensation and treatment for subjects who are harmed as a result of participating in research must be ensured (World Medical Association, 2008).” The Presidential Commission for the Study of Bioethical Issues has made recommendations regarding the need for compensation for research‐related injuries, but there has been no clear response from the federal government (Presidential Commission for the Study of Bioethical Issues, 2011).

^2^
A higher‐education institution that performs both covered and noncovered functions may choose to be a hybrid entity under the HIPAA (45 CFR §164.103). To become a hybrid entity, an institution must define and designate its health care component, separate from other components that perform noncovered functions, and the HIPAA Privacy or Security Rule requirements would apply only to the health care component.

^3^
Under the Health Information Technology for Clinical and Economic Health (HITECH) Act, part of the American Recovery and Reinvestment Act of 2009, State Attorneys General has the authority to bring civil actions on behalf of state residents for violations of the HIPAA Privacy and Security Rules. State Attorneys General can obtain damages on behalf of state residents or to enjoin further violations of the HIPAA Privacy and Security Rules (U.S. Department of Health and Human Services (HHS), 2017b).

^4^
Certificate of Confidentiality (CoC) is issued to persons engaged in biomedical, behavioral, clinical, or other research—funded wholly or in part by NIH, in which identifiable, sensitive information is collected. CoCs protect the privacy of research subjects by prohibiting disclosure of identifiable, sensitive research information to anyone not connected to the research except when subject consents or in a few other specific situations (Office of Human Research Protections (OHRP), 2003).

^5^
The standard INDI data sharing policy is Creative Commons, Attribution‐NonCommercial‐ShareAlike license, but some datasets in the INDI are shared under more or less restrictive licenses (e.g., Creative Commons, Attribution‐Non‐Commercial license in the ADHD‐200 or Open Data Commons Public Domain Dedication and License (PDDL) in the Max Planck Institute Leipzig Mind‐Brain‐Body dataset) or under their own Data Use Agreement (e.g., Anatomical Tracings of Lesions After Stroke (ATLAS)).

^6^
The data collected as part of Lifespan HCP project are housed in the National Institute of Mental Health Data Archive (NDA) and access to these data is governed by the Adolescent Brain Cognitive Development (ABCD) (National Institute of Mental Health Data Archive (NDA), 2019).

^7^
The Patient Protection and Affordable Care Act (ACA) also contains provisions that prevent health insurers from discriminating against an individual (e.g., denying coverage or charging a higher premium) on the basis of an individual's participation in medical research. Yet here medical research is limited to a clinical trial conducted in relation to the prevention, detection, or treatment of a life‐threatening disease or condition, such as a cancer. In addition, the ACA does not prohibit “discrimination based on the disclosure of information gathered during research, but merely discrimination based on enrollment (Arias & Karlawish, 2014).”

^8^
Other scholars have proposed legislation akin to GINA regarding neuroscience data under different perspectives. For example, regarding the risks of using neuroscience data to compel suspects or defendants to produce self‐incriminating evidence in criminal cases by decoding inner thoughts or memories, Farahany suggests adopting a GINA‐like statutory approach for the protection of mental privacy, or cognitive liberty, against unwarranted government intrusions (Farahany, 2012).
